# Augmenting GPS IWV estimations using spatio-temporal cloud distribution extracted from satellite data

**DOI:** 10.1038/s41598-018-33163-x

**Published:** 2018-10-03

**Authors:** Anton Leontiev, Yuval Reuveni

**Affiliations:** 1 Department of Electrical Engineering, Ariel University, Ariel, Israel; 2 Department of Physics, Ariel University, Ariel, Israel; 3 Eastern R&D Center, Ariel, Israel; 4 School of Sustainability, Interdisciplinary Center (IDC) Herzliya, Herzliya, Israel

## Abstract

Water vapor (WV) is the most variable greenhouse gas in the troposphere, therefore investigation of its spatio-temporal distribution and motion is of great importance in meteorology and climatology studies. Here, we suggest a new strategy for augmenting integrated water vapor (IWV) estimations using both remote sensing satellites and global positioning system (GPS) tropospheric path delays. The strategy is based first on the ability to estimate METEOSAT-10 7.3 *µm* WV pixel values by extracting the mathematical dependency between the IWV amount calculated from GPS zenith wet delays (ZWD) and the METEOSAT-10 data. We then use the surface temperature differences between ground station measurements and METEOSAT-10 10.8 *µm* infra-red (IR) channel to identify spatio-temporal cloud distribution structures. As a last stage, the identified cloud features are mapped into the GPS-IWV distribution map when preforming the interpolation between adjusted GPS station inside the network. The suggested approach improves the accuracy of estimated regional IWV maps, in comparison with radiosonde data, thus enables to obtain the total water amount at the atmosphere, both in the form of clouds and vapor. Mean and root mean square (RMS) difference between the GPS-IWV estimations, using the spatio-temporal clouds distribution, and radiosonde data are reduced from 1.77 and 2.81 kg/m^2^ to 0.74 and 2.04 kg/m^2^, respectively. Furthermore, by improving the accuracy of the estimated regional IWV maps distribution it is possible to increase the accuracy of regional Numerical Weather Prediction (NWP) platforms.

## Introduction

Water vapor (WV) is one of the main greenhouse gases which has a significant contribution related to climate and weather changes^1,2^. Due to its large concentration in the atmosphere^3^, WV plays a key role in the greenhouse effect as it repetitively cycles through evaporation and condensation, transporting heat energy around the Earth and between the surface and the atmosphere^4^. While WV in the atmosphere contracts the short wavelength radiation, emitted from the sun, as it propagates through the atmosphere, it also traps the long wavelength radiation emitted from the Earth’s surface^5^. This confined radiation causes temperatures to increase, which in turn enable the air to sustain a larger amount of WV, thus magnifying the greenhouse effect^2^. Investigating WV distribution and motion in the lower part of the atmosphere is necessary for climatology and meteorology studies^6^, as it also plays a major part in determining climate sensitivity. However, our current ability to constantly monitor WV distribution changes at high spatial resolution still remains insufficient^3,7^.

There are several methods for estimating the amount of WV in the troposphere. The most common ones use radiosondes^6–8^, different techniques of GPS tropospheric path delays^2,9–16^, or remote sensing measurements from satellites such as the METEOSAT series^17–19^. Recently, *Leontiev and Reuveni*^20^ developed a technique for combining GPS tropospheric zenith path delays with near-real time METEOSAT-10 pixel intensity values (using 7.3 $\mu {m}$ and 10.8 $\mu m$ WV and surface temperature channels, respectively), in order to obtain absolute IWV (kg/m^2^) distribution maps. The results showed good agreement between the estimated IWV values obtained from their triangulation strategy, based solely on GPS Zenith Total Delays (ZTD) and METEOSAT-10 surface temperature data (10.8 $\mu m$ channel), compared with *in-situ* IWV measurements from radiosondes.

The suggested technique is also capable of determine the mathematical dependency between METEOSAT-10 7.3 µm WV pixel values and the estimated GPS-IWV absolute amount^20^. The main advantage of using the converted METEOSAT-10 7.3 $\mu m$ WV pixel values is that it can potentially be used for producing WV distribution maps using the METEOSAT-10 data and a small number of GPS station data. The main disadvantage of this technique however, is the uncertainty regarding METEOSAT-10 7.3  $\mu m$ extremely low (and high) intensity pixel values. Low intensity pixel value usually means that the amount of water in the surrounding area is very high, and most likely it is due to clouds (or moist air areas) which absorbs the Earth’s surface emitted radiation beneath. Therefore, this might introduce a bias to the mathematical dependency between the METEOSAT-10 7.3 $\mu m$ WV channel and the GPS-IWV estimations. The two different approaches for deriving regional WV distribution maps; triangulating IWV estimations using GPS ZWD combined with METEOSAT-10 surface temperature data (10.8 $\mu m$ IR channel), or alternatively converting METEOSAT-10 WV intensity pixel values (7.3  $\mu m$ WV channel) using a mathematical dependency to a known estimated GPS WV value, were both compared with radiosonde IWV absolute values. It was shown that triangulating IWV data obtained from a network of GPS stations combined with surface temperature estimations is more accurate and detailed than the IWV data obtained from Meteosat-10 7.3 $\mu m$ calibrated observations^20^.

Using the above mentioned techniques, it is possible to produce adequate WV distribution maps for investigating spatio-temporal WV motions. However, this task still remains challenging due to the WV large variability^21^ and the ability of water to migrate between three different phases along the atmospheric temperatures range^22^. Therefore, when investigating the total water content spatio-temporal distribution and motion, it is necessary to take into account both WV and clouds, which occasionally consists of liquid water and ice drops.

Furthermore, due to the short-term temporal and spatial variability of WV, it is difficult to properly model accurate and precise high spatial resolution WV distribution in real-time. There are some models, which only produce WV amount at the atmosphere (e.g. Weather Research and Forecasting (WRF) model, http://www.wrf-model.org/index.php) but do not take into accounts clouds and other hydrometeors^23^. The WRF model, for example, allows to assimilate WV distribution maps similar to the ones obtained using interpolated IWV values derived from GPS ZWD^20^. However, as mentioned above, when interpolating the absolute IWV values from a network of GPS stations, clouds also contribute to the total atmospheric IWV content, and consequently should be taken into account while interpolating between the network points. The suggested approach will be extremely useful for the Middle East dry areas, because of the relative high humidity and WV condensation ability with additional rain, which is very important for agriculture and other human activities.

## Methodology for IWV estimation from GPS

The GPS data used in this study were processed separately for each day using the JPL GIPSY-OASIS precise point positioning (PPP) software and products. A 7° minimum elevation cut-off for the satellite observations was applied along with the Vienna Mapping Function 1 (VMF1)^24^. Zenith hydrostatic delay (ZHD) values from the VMF1 Grid were used every 6 hours. The GIPSY-OASIS software considers the tropospheric zenith delay and gradients as stochastic parameters to allow time varying behavior. Stochastically time varying parameters are assumed to be constant within each time step, but are allowed to change from one time step to another. After a measurement has been processed (and the parameter estimation had been updated), a time update is performed, adding process noise to the parameter uncertainties in order to simulate unmodeled or mismodeled effects^25–27^. Once the ZWD value is obtained for a specific time interval (i.e. 5 minutes) the IWV can be easily calculated using the surface temperature (Bevis *et al*., 1992). For a more detailed description of the methodology, as well as the exact values for tropospheric parameters, see *Leontiev and Reuveni*^20^.

### Estimating GPS IWV distribution maps

**Figure 1:**
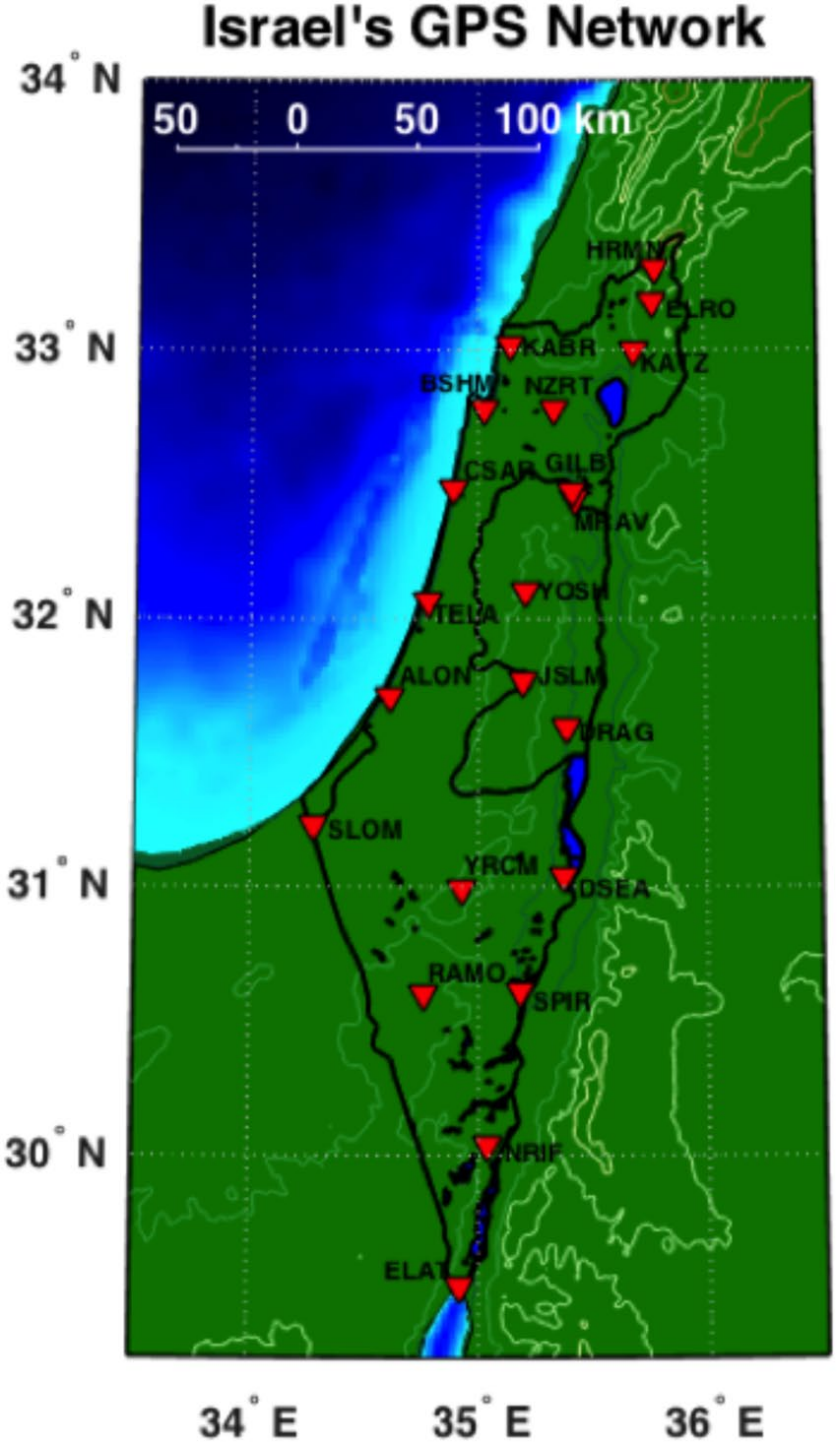
Israel’s SOI-APN GPS network. The network is maintained by the Survey of Israel (MAPI) and is consisted of 24 permanent geodetic GPS receivers.

**Figure 2:**
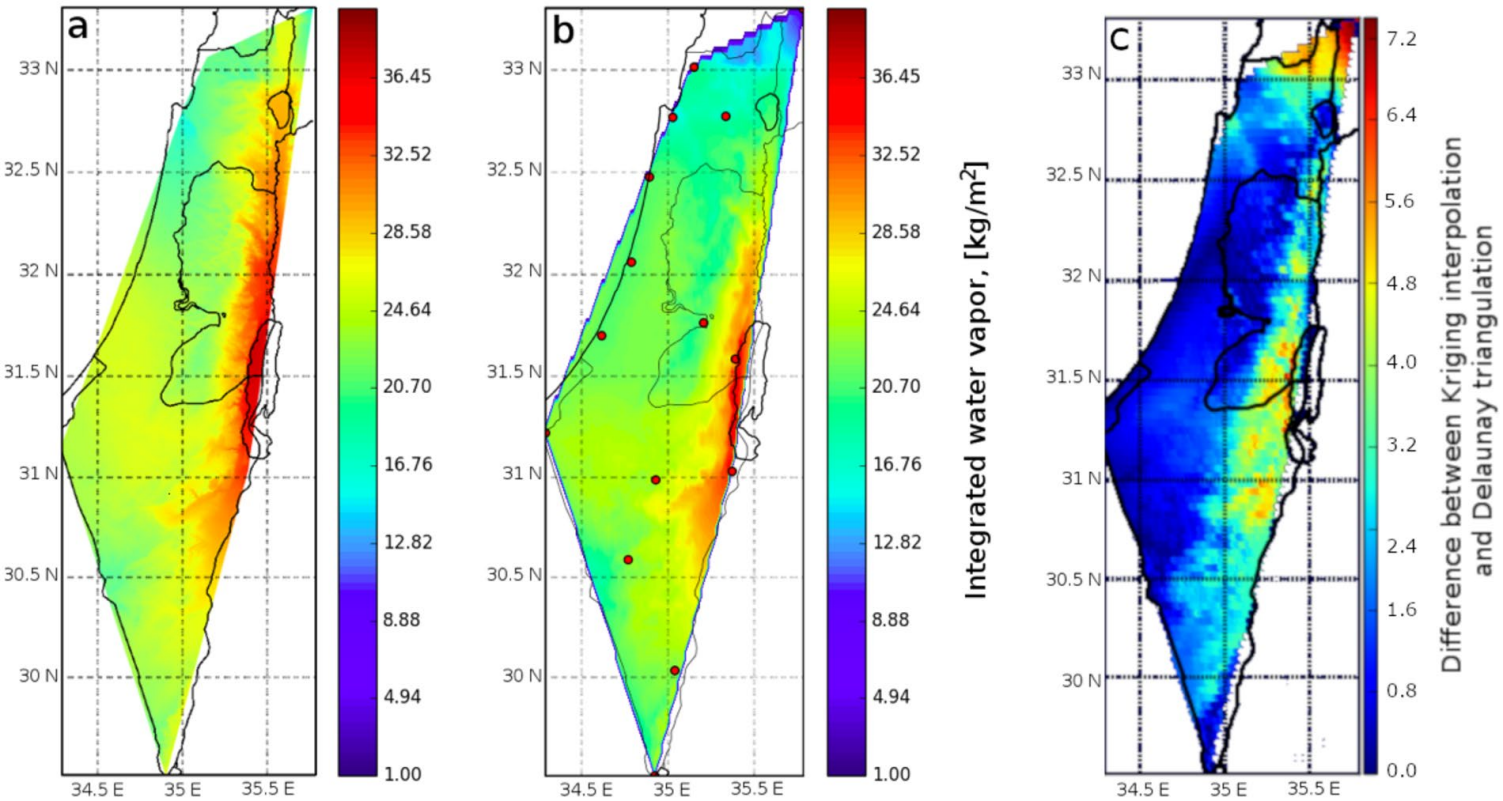
Example for Kriging interpolation on the left panel (**a**) and Delaunay triangulation on the middle panel (**b**) produced for 21.08.2015, 12:00 UT. The difference between the two methods is presented on the righthand panel (**c**) where mean and RMS difference are 1.85 and 2.33 kg/m^2^, respectively. The red circles on the middle panel (**b**) represent Israeli GPS stations which were used.

**Figure 3:**
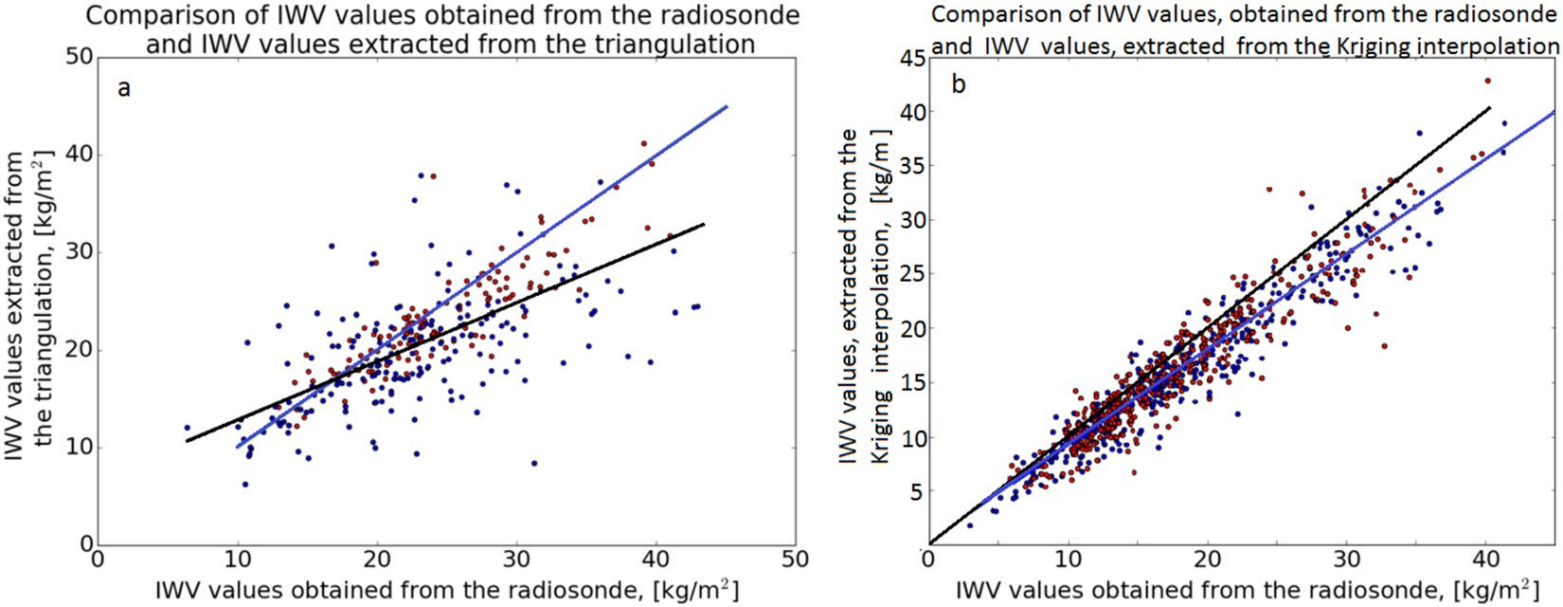
Comparison between IWV values obtained from radiosonde data versus the Delaunay triangulation (**a**) and Kriging interpolation (**b**). The blue lines shows linear dependency with correlation coefficient 0.45 (**a**) and 0.9 (**b**) obtained using the least square method between the two data sets and the radiosonde data. The black line shows where both of sets are equal. Red dots correspond to daytime measurements (12:00 UT), and blue dots correspond to nighttime measurements (00:00 UT). (**a**) For the Delaunay triangulation mean and RMS values are 2.06 and 5.69 kg/m^2^, respectively. (**b**) For the Kriging interpolation mean and RMS values are 1.77 and 2.81 kg/m^2^, respectively.

Once ZWD estimated values are obtained from the entire GPS ground-station network, a distribution map can be constructed using different interpolation methods. Here, we considered using either the Delaunay triangulation^28^ or Kriging interpolation^29^ applied with the IWV values obtained from the survey of Israel-active permanent network (SOI-APN) GPS network data (Fig. 1). A comparison between the two is shown in Fig. 2, where the Kriging interpolation yields smoothest (yet more detailed) results in comparison with the Delaunay triangulation technique. Mean and root mean square (RMS) difference between the two are 1.85 and 2.33 kg/m^2^, respectively. The Kriging interpolation was applied along the area of 29–35°N and 33–36°E, which covers the entire area of Israel and several parts of Lebanon, Syria, Jordan and Egypt. GPS ZWD data for constructing the values of WV were taken only from the SOI-APN GPS network since GPS data from other countries were not available. We used standard Python libraries when taking earth topography into account (instead of interpolating across terrain features) while applying Delaunay triangulation or Kriging interpolation. Furthermore, the best way to determine the accuracy of regional IWV map (constructed from triangulating all available GPS data) is to compare the IWV values above the exact location where the radiosonde observations are taken (i.e. at Bet Dagan site). On this basis, we produced 500 consecutive IWV maps for 2015–2016 using both Delaunay triangulation and Kriging interpolation, and compared the values at each map above Bet Dagan to radiosonde IWV observations (Fig. 3). The correlation coefficient, using least square method, between the Kriging interpolation and radiosonde data is R^2^ = 0.9, where the mean and RMS difference are 1.77 and 2.81 kg/m^2^, respectively. The correlation coefficient between the Delaunay triangulation and radiosonde data is R^2^ = 0.45, where the mean and RMS difference are 2.06 and 5.69 kg/m^2^, respectively.

### Augmenting GPS IWV estimations using cloud distribution extracted from METEOSAT-10 satellite data

Using the technique developed by *Leontiev and Reuveni*^20^ for extracting tropospheric zenith path delays combined with near-real time METEOSAT-10 Water Vapor (WV) and surface temperature pixel intensity values, it is possible to produce WV distribution maps over the entire Israel area. However, the interpolation does not take into account clouds, which are formed between GPS stations inside our network. Using our results for translating Meteosat-10 7.3 *µm* WV pixel values into total amount of WV^20^, we can add the “cloudy” values of WV to the interpolated GPS maps and obtain even more realistic and accurate maps.

There are different types of clouds at the atmosphere, which are formed at different heights range. There are three types of high clouds (cirrus, cirrostratus and cirrocumulus), middle height clouds (altostratus, altocumulus and nimbostratus) and four types of low height clouds (cumulus, stratus, cumulonimbus and stratocumulus)^30^. The low height clouds make the most significant contribution to liquid water content, where their density can vary from 1 g/m^3^ up to 3 g/m^3^, while the density of the middle and high-altitude clouds reach less than 0.45 g/m^3 ^^31^. Assuming that the height of the upper border of cumulonimbus cloud can reach 10 km, it will contribute up to an additional 30 kg/m^2^ into the total water content in addition to WV contents. A large number of cloud parameters may be obtained using the NWCSAF (http://www.nwcsaf.org/) service and products, such as the cloud upper border height, position, content (if it is water or ice cloud) as well as total water vapor content at a clear sky conditions. Thus, using these products, it is possible to estimate the current state of weather, cloud coverage and even make a short weather forecast. However, the NWCSAF platform does not provides the total water contents above the cloudy area, from which we can gain a better understanding regarding the movement of the total water contents across the Earth’s atmosphere, rather than only the WV contents itself.

The Meteosat-10 channels, which allows to estimate WV content in the atmosphere, indicate the amount of thermal energy absorbed during the way from the Earth surface to satellite by the atmospheric water content, which appears both as liquid (i.e. clouds) and/or gas (i.e. WV). High absorption usually means larger amount of water. Here, we suggest using Meteosat-10 WV channels to estimate spatio-temporal clouds distribution based on analyzing absorption features within each satellite image. Yet, it is a challenging task since high absorption merely indicates that there is large amount of WV in the atmosphere. Therefore, so it is practically impossible to determine whether the absorption is caused by clouds or by highly saturated air under clear sky conditions. In the case of estimating WV distribution using the interpolation procedure explained above, the interpolation succeeds to reveal moist air areas, but fail to reveal any form of cloud while interpolating between the network points. Using the approach described below, we show that it is possible to estimate clouds locations by exploiting the absorption features from Meteosat-10 surface temperature and WV images, and take them into account while estimating the total water content while constructing the GPS-IWV map distribution.

**Figure 4:**
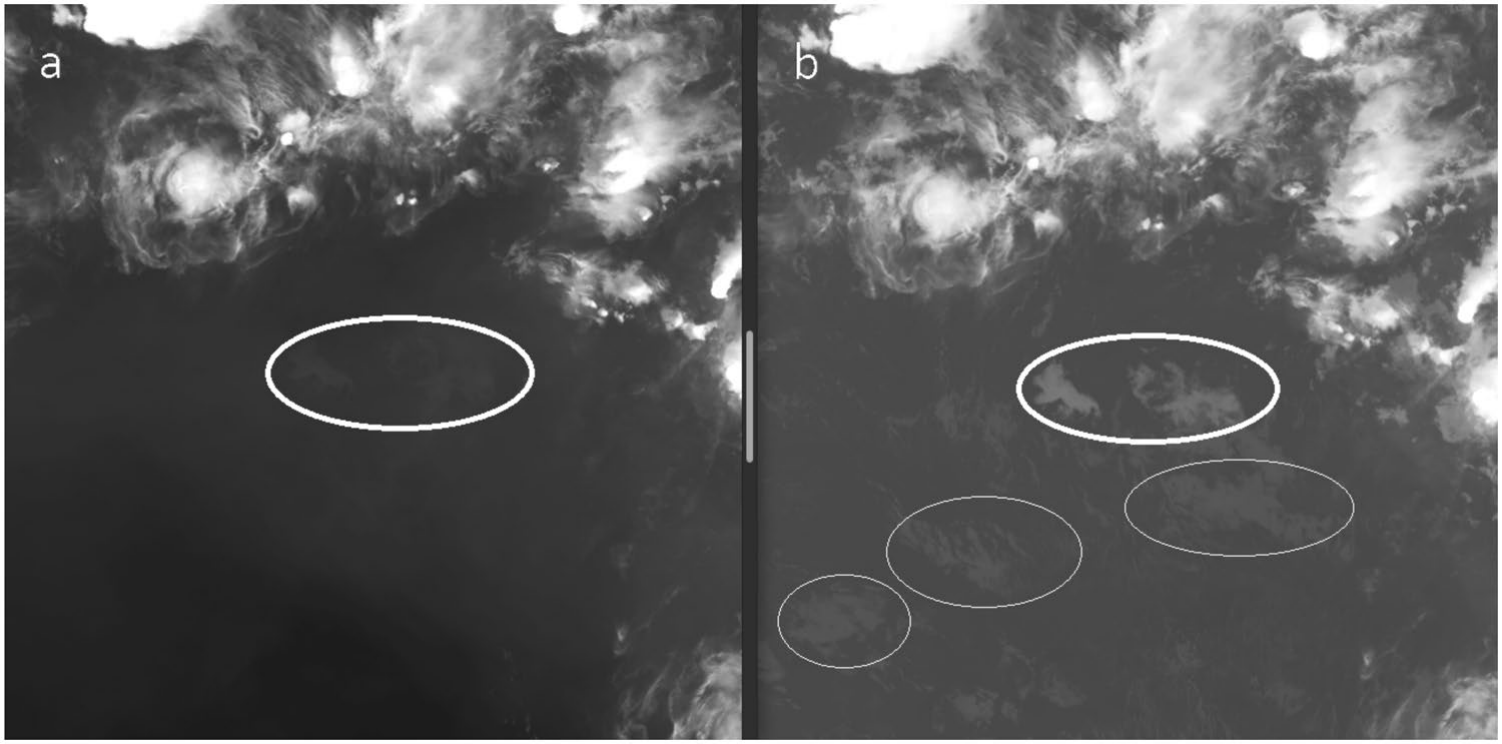
Difference between Meteosat-10 7.3 *μm* WV (**a**) and 10.8 *μm* IR (**b**) channels with respect to the ability of revealing liquid water (i.e. clouds structures). Both channels succeeds in revealing certain type of clouds (thick line ellipses), but additional clouds structures are revealed only in 10.8 *μm* IR channel (thin line ellipses). Copyright 2018 EUMETSAT.

In general, a clear atmosphere is transparent to IR radiation at what is known as IR atmospheric windows^32^ with typical wavelengths ranging between 8–14 *μm*. Meteosat-10 utilizes two channels with such wavelengths, 10.8 and 12 *μm*. Here, the 10.8 *μm* channel is used for estimating the surface temperature reference values. Since clouds are colder than the Earth surface, at 10.8 *μm* band they appear as areas with lower temperatures. Therefore, calculating the differences between the temperatures estimated by the Meteosat-10 10.8 *μm* IR channel and Israeli meteorological service (IMS) ground stations reference values, can help us define an area which the IR radiation, emitted from the Earth’s surface, is absorbed solely by liquid water. This is opposed to the Meteosat-10 7.3 *μm* WV channel where pixels with lower temperatures might indicate either moist air region (i.e. WV) or liquid water (i.e. clouds). A good example which demonstrate this distinction between the two band is shown in Fig. 4, where the 10.8 *μm* IR channel succeeds in revealing all clouds structures, but the 7.3 *μm* WV channel partially reveals only one cloud structure above the same area.

Once a threshold for the temperature differences between the Meteosat-10 10.8 *μm* IR channel and IMS ground stations is set, it is possible to extract the clouds structure using the exact pixel coordinates within the Meteosat-10 images which exceeds the temperature difference threshold. In comparison with ground surface temperature (such as the IMS ground stations measurements), Meteosat-10 10.8 *μm* IR channel underestimates temperatures during day times and vice versa during night times^18^. The maximal difference between ground and remote sensed temperature measurements is less than 5 °K and approximately changes from ΔT = −5 up to 5 °K, depending on the time of day^18^. Using the difference between the two measured temperatures, it is possible to estimate not only the fact that there are cloudy or clear sky conditions, but possibly also the actual cloud top heights (assuming we know the surface and cloud top temperatures along with an estimated temperature gradient).

**Figure 5:**
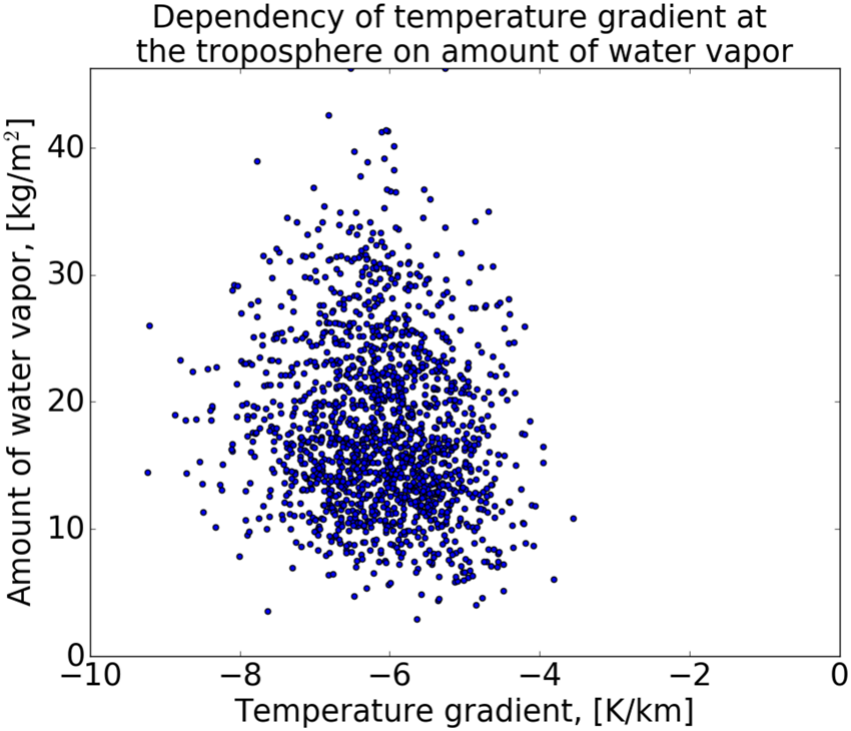
Temperature gradient as function of WV content in the air, extracted from radiosonde data over one year. Values varies between −4 to −8 °K/km, therefor we set an average value in between, Γ = −5 °K/km.

In order to gain the ability to extract the temperature differences between the IMS surface temperature measurements and Meteosat-10 10.8 *μm* IR channel, IMS point measurements should be interpolated into a distribution map. For estimating the absolute surface temperature at higher spatial resolution, we applied the Kriging interpolation with the IMS surface temperature point measurements while taking Earth topography into account. Since most of the WV is concentrated in the lower part of the troposphere with a scale height of ≈3 km^22^, it is reasonable to assume that the total amount of WV (excluding clouds) lies under an altitude of 9 km. The amount of WV above this height is *e*^3^ *≈* 20 times lower, i.e. 95% of the total WV amount is concentrated below an altitude of ≈9 km. Furthermore, at this height range the tropospheric temperature gradient changes linearly with height, and has a weak dependency with air moisture, while it changes at a range of Γ = −8 to −4 °K/km (Fig. 5). A mean reasonable value for the temperature gradient can be set to −5 °K/km. Similar values were also obtained by previous studies^33^.

**Figure 6:**
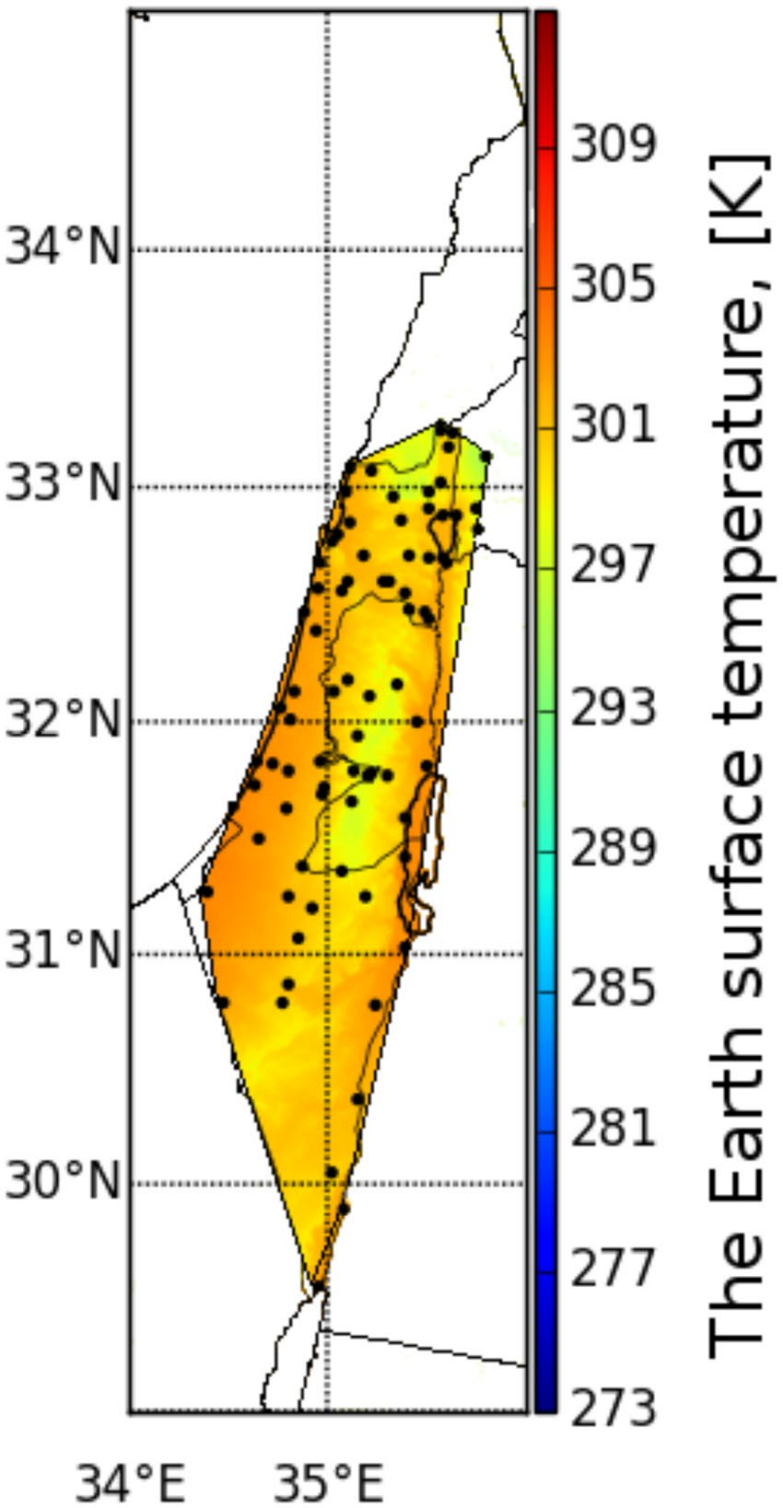
Example for the temperature interpolation using Kriging interpolation over Israel area extracted from the IMS meteorological stations temperature data (indicated as black points).

The procedure for interpolating the temperature values is similar to the WV interpolation, where every available IMS meteorological ground station temperature is scaled to sea level (sl), using the relation:

1 $$T={T}_{sl}+h\Gamma {,}$$

where *Γ* is the temperature gradient and *h* is an altitude above (with “+”) or below (with “−”) sea level height. Thus, these values should be all interpolated in order to obtain the adequate spatial temperature distribution at sea level height. Aa a last step, after applying the interpolation to sea level, the interpolated temperature field is then scaled separately to terrain elevation using equation (1) and a digital elevation model such as SRTM3. For example, for Israel area the temperatures at the Samarian hills or the Golan Heights will be lower than the sea level temperature as pressure decreases with increasing altitude, while at the Jordan Valley and the Dead Sea regions it will be higher than sea level temperatures because they lie below sea level (where the pressure is higher). Figure 6 shows a good example for the temperature interpolation result. Normally, surface temperatures above land are higher than temperatures above the sea during daytime and vice versa during nighttime. This is due to the fact that water gain higher heat capacity compared with the ground, which translates to slower heating during daytime and slower cooling during nighttime in comparison with the ground. However, as it can be seen from Fig. 6, the extracted Meteosat-10 surface temperatures at the Gulf of Aqaba, during daytime, are much higher than its surrounding ground temperature. This inaccuracy appears due to the fact that the interpolation scheme does not take into account the type of surface (i.e. land or sea) and considers every point in the grid as land.

After interpolating the temperature field, it is possible to calculate the difference between the temperatures obtained from the two sources (IMS ground surface data and Meteosat-10 10.8 *μm* channel), and reveal any cloud structure with its exact location. A fair amount of efforts show that a mean temperature difference between Meteosat-10 derived temperature and IMS interpolated temperature field, equal or bigger than 2 °K can confidently indicate that a cloud structure is found. After revealing the clouds spatial distribution, it is possible to calculate its WV content using the mathematical dependency between Meteosat-10 7.3 *μm* pixels value and the absolute GPS-IWV amount^20^. As mentioned above, the Meteosat-10 land/sea daytime and nighttime under- and overestimation surface ground temperature should be taken into account when extracting Meteosat-10 surface temperature values. One more problem with augmenting the interpolated WV distribution maps with Meteosat-10 satellite imagery is that after calculating the difference between the two data sources, the interpolation scheme considers ground water sources, which are usually colder than the ground surface, as clouds. For example, the Dead Sea and lake Kinneret are not taken into account as ground water objects, and due to the procedure described above their spatial area is replaced by the satellite-derived WV values. It should be pointed out that the suggested strategy (using the Meteosat-10 data) will be sensitive only for clouds with spatial horizontal scale larger than 5 km. This restriction is caused by the Meteosat-10 sensor’s resolution, as mentioned earlier, which varies depending on the longitude and latitude. Each pixel of Meteosat-10 image covers an area ranging from 3 × 3 km^2^ per pixel near the equator to 11 × 11 km^2^ per pixel near the Earth’s limb. For Israel, each pixel covers an area with a 5 × 5 km^2^ resolution.

## Results

**Figure 7:**
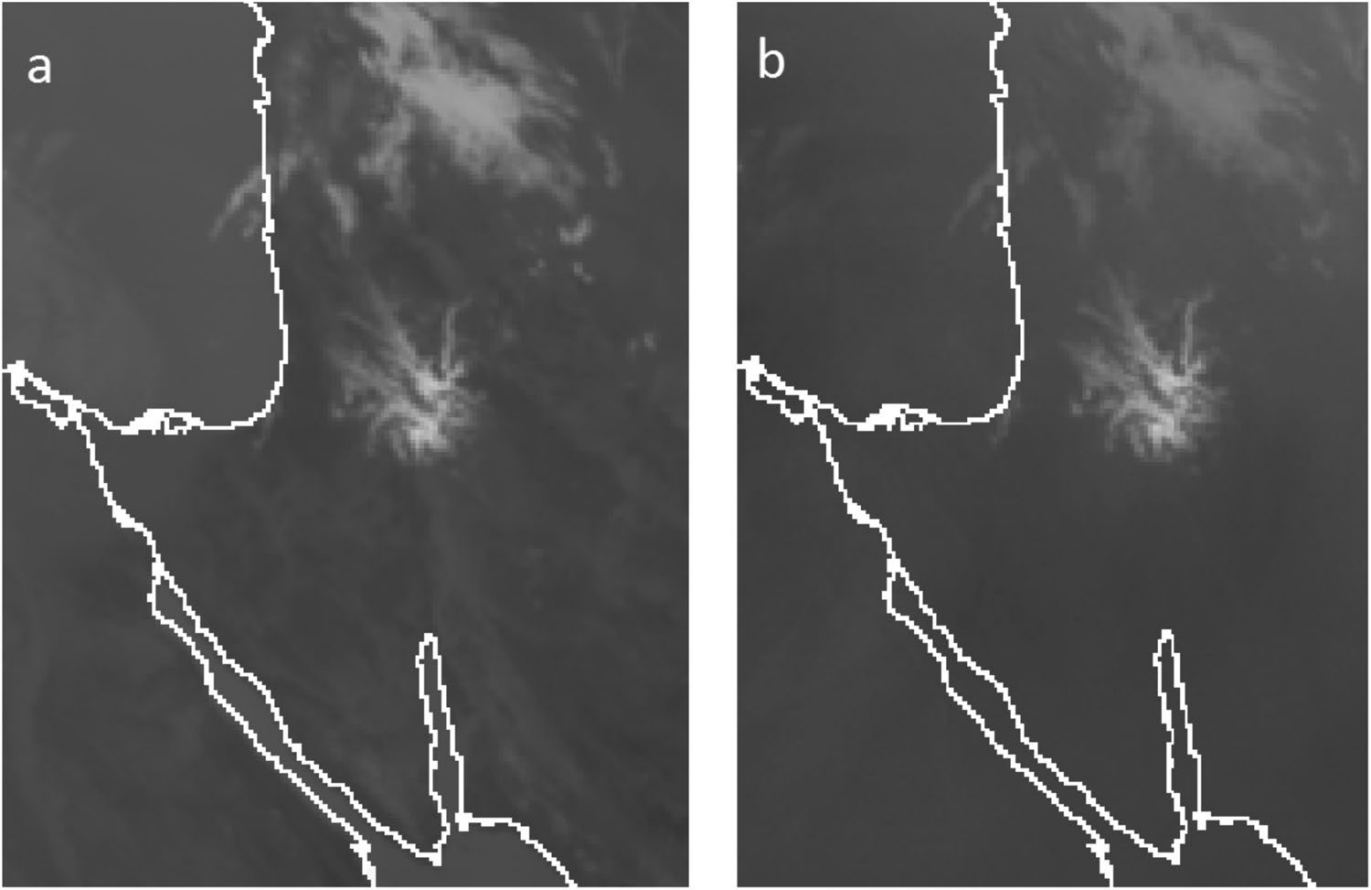
Meteosat-10 images for Israel area, obtained from (**a**) 10.8 *µm* surface temperature channel and (**b**) 7.3 *µm* WV channel. Light pixels in (**a**) correspond to low temperature, and light pixels in (**b**) correspond to either moist area or clouds. For the 10.8 *µm* surface temperature channel, clouds are revealed as light gray structures (i.e. areas of low temperature). Copyright 2018 EUMETSAT.

Using the strategy described above, it is possible to estimated different cloud parameters such as position, cloud top heights and water content during different seasons. As such, constructing WV maps using a combination between the interpolation scheme and cloud distribution features, enable to produce enhanced maps of the IWV over Israel area, which varies significantly both diurnally and seasonally. For example, during spring, summer and autumn periods, most of the water in the atmosphere is distributed as vapor and clouds rarely appear. Thus, values of WV can reach up to 30 kg/m^2^ and more, but during winter, which is the rainy season, most of water exist in form of clouds and the IWV values are small, varying between 5–15 kg/m^2^, while all the remaining water exists as clouds. Figure 7 shows an example of clouds captured from 7.3 and 10.8 *µm* Meteosat-10 channels, which corresponds to the WV and surface temperature, respectively. For revealing any cloud structure (i.e. pixels location), we compared the temperature measured by the METEOSAT-10 (Fig. 7a) with the IMS interpolated temperatures. If the difference between the interpolated IMS temperature and METEOSAT-10 observations (10.8 *µm* WV channel) is larger than 2 °K, we considered this area as cloud. As can be seen from a visual comparison to the 7.3 *µm* WV channel (Fig. 7b), this approach manage to first distinguish between moist area and actual clouds, while identifying the exact cloud structure and location.

**Figure 8:**
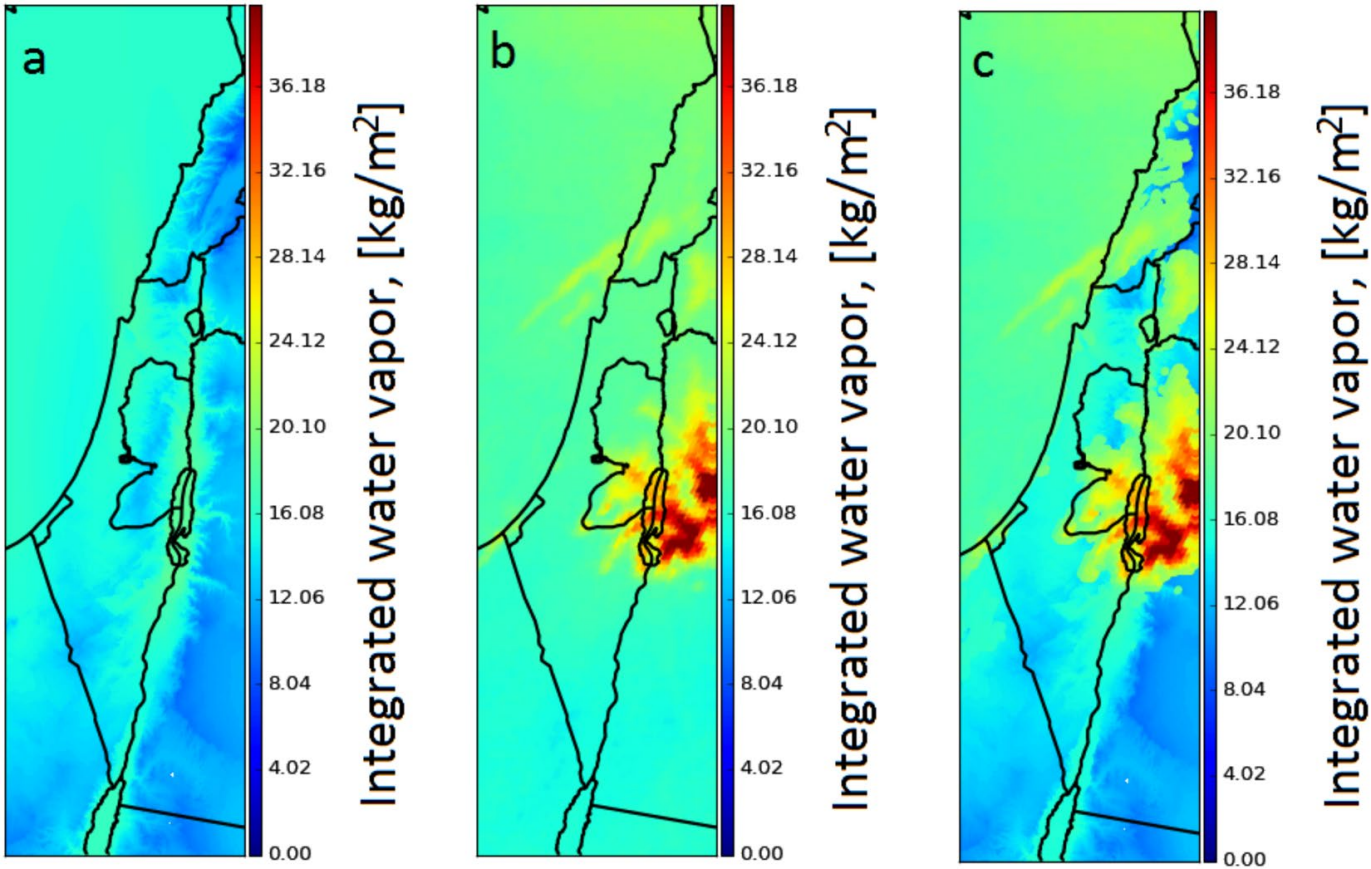
Interpolated GPS-IWV field augmented with cloud features. (**a**) Kriging interpolation using IWV-GPS estimations. (**b**) WV distribution, which corresponds to Fig. 7b (7.3* µm* WV channel). (**c**) combining (**a**) and the cloud features based on the temperature differences between IMS interpolated temperature and 10.8 *µm* temperature channel (Fig. 7a).

**Figure 9:**
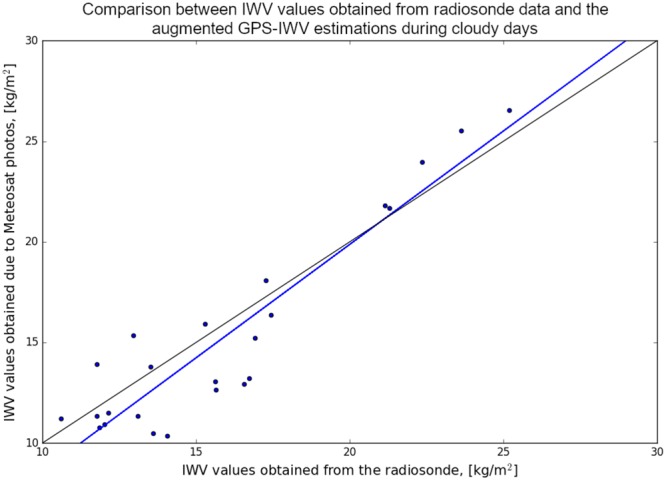
Comparison between IWV values obtained from radiosonde data versus the GPS-IWV values, using the spatio-temporal clouds distribution, for days where clouds were apparent above the IMS radiosonde launch site (above Bet Dagan). The blue lines shows linear dependency with correlation coefficient 0.93, obtained using the least square method between the GPS-IWV data set and the radiosonde data. The black line shows where both of sets are equal. Mean and RMS difference between the two data sets were reduced from 1.77 and 2.81 kg/m^2^ to 0.74 and 2.04 kg/m^2^, respectively.

After extracting the pixels location which corresponds to a cloud structure, we can use the WV values dependency, obtained from IWV-GPS and Meteosat-10 pixel values, to adjust the pixel values to an actual IWV values. We can then interpolate the GPS-IWV field augmented with these cloud features, to obtain a better assessment and more detailed information in comparison with WV distribution maps extracted solely from satellite images or GPS ZWD data (Fig. 8). In order to quantify how well the suggested augmented strategy perform, we compared the GPS-IWV values, using the spatio-temporal clouds distribution, and radiosonde data, for days where clouds were apparent above the IMS radiosonde launch site (above Bet Dagan). Mean and RMS difference between the two data sets were reduced from 1.77 and 2.81 kg/m^2^ to 0.74 and 2.04 kg/m^2^, respectively (Fig. 9).

## Discussion

Based on our suggested strategy described above, the obtained results indicate that it is possible to augment interpolated GPS IWV maps using remote sensing satellite data, such as the Meteosat-10 satellite, in order to introduce spatio-temporal cloud distribution while preforming the interpolation between adjusted GPS station inside the network. It is also shown, that the temperature distribution over a specified area might be obtained using Kriging interpolation. The main advantage of interpolating the temperature values is gaining high spatial resolution and precision compared with the limited precision of ground temperature measurements. On the other hand, this technique is not sensitive enough to different type of surfaces. As mentioned above, the temperatures over the sea during daytime are lower than the ground temperature, however the interpolation procedure considers the sea and ground surfaces as the same type, which in turn leads to incorrect estimation of the water temperatures. As it was mentioned above, this fact evokes some challenges while combining Meteosat-10 data with Kriging interpolation data, because in some cases water-filled areas (e.g., the Dead Sea and lake Kineret) might be considered by this technique as cloud. In order to avoid this issue, we compare the satellite-derived values with the interpolated values and leave the higher one. Correct estimation of water temperatures might be achieved with a large number of coastline temperature measurements in order to properly estimate the borders between the ground and sea. This inaccuracy of temperatures estimation due to the interpolation procedure does not have any influence what so ever on the ability to correctly revealing the cloud structure and features during morning or evening times, where the temperatures are approximately equal, however this equilibrium normally exists for a short time periods and strongly depends on the weather conditions. Therefore, due to this large uncertainty, another technique is needed for verifying any cloud structure or feature above sea surface areas.

## Conclusions

In this study, we present an enhance strategy for estimating GPS-IWV distribution maps augmented by spatio-temporal cloud distribution extract from satellite data. The proposed technique is based on a previous work which combine GPS-IWV estimation along with Meteosat-10 satellite image data, and might be applied for any region above the Earth after proper calibration of the Meteosat-10 data. Furthermore, the suggested technique allows to estimate clouds size, form, location, top heights and water content, at spatial horizontal scale larger than 5 km due to the spatial resolution confinement of the Meteosat-10 sensors (5 × 5 km^2^ per pixel). In addition, this technique also allows to provide a more accurate GPS IWV distribution maps, which include both satellite and GPS ZWD data while harnessing the advantages of these two methods, in order to properly evaluate the IWV amount at cloudy conditions when preforming the interpolation between adjusted GPS station inside the network. Mean and RMS difference between the augmented GPS-IWV and radiosonde data were reduced from 1.77 and 2.81 kg/m^2^ to 0.74 and 2.04 kg/m^2^, respectively. Furthermore, using spatio-temporal cloud distribution obtained form Meteosat-10 data combined with GPS derived IWV values, it is possible to produce IWV distribution maps every 15 minutes to provide water content predictions or study processes which already occurred.
